# suPAR in the assessment of post intensive care unit prognosis: a pilot study

**DOI:** 10.5935/0103-507X.20180062

**Published:** 2018

**Authors:** Joana Silvestre, Luis Coelho, João Gonçalves Pereira, Vitor Mendes, Camila Tapadinhas, Pedro Póvoa

**Affiliations:** 1 Unidade de Terapia Intensiva Polivalente, Hospital São Francisco Xavier, Centro Hospitalar Lisboa Ocidental - Lisboa, Portugal.; 2 . Centro de Estudos de Doenças Crônicas, Faculdade de Ciências Médicas, Universidade Nova de Lisboa - Lisboa, Portugal.

**Keywords:** Receptors, urokinase plasminogen activator, C-reactive protein, Biomarkers, Prognosis

## Abstract

**Objective:**

To determine the performance of soluble urokinase-type plasminogen activator receptor upon intensive care unit discharge to predict post intensive care unit mortality.

**Methods:**

A prospective observational cohort study was conducted during a 24-month period in an 8-bed polyvalent intensive care unit. APACHE II, SOFA, C-reactive protein, white cell count and soluble urokinase-type plasminogen activator receptor on the day of intensive care unit discharge were collected from patients who survived intensive care unit admission.

**Results:**

Two hundred and two patients were included in this study, 29 patients (18.6%) of whom died after intensive care unit discharge. Nonsurvivors were older and more seriously ill upon intensive care unit admission with higher severity scores, and nonsurvivors required extended use of vasopressors than did survivors. The area under the receiver operating characteristics curves of SOFA, APACHE II, C-reactive protein, white cell count, and soluble urokinase-type plasminogen activator receptor at intensive care unit discharge as prognostic markers of hospital death were 0.78 (95%CI 0.70 - 0.86); 0.70 (95%CI 0.61 - 0.79); 0.54 (95%CI 0.42 - 0.65); 0.48 (95%CI 0.36 - 0.58); and 0.68 (95%CI 0.58 - 0.78), respectively. SOFA was independently associated with a higher risk of in-hospital mortality (OR 1.673; 95%CI 1.252 - 2.234), 28-day mortality (OR 1.861; 95%CI 1.856 - 2.555) and 90-day mortality (OR 1.584; 95%CI 1.241 - 2.022).

**Conclusion:**

At intensive care unit discharge, soluble urokinase-type plasminogen activator receptor is a poor predictor of post intensive care unit prognosis.

## INTRODUCTION

In-hospital death following intensive care unit (ICU) discharge has been estimated to be 5% - 27%, and nearly 10% of discharged patients require ICU readmission.^(1-4)^ Despite improvements in ICU care quality and widespread utilization of step-down units over the last decades, a significant number of patients still die in the hospital following successful ICU discharge;^(5)^ therefore, adequate evaluation is necessary to identify individuals at high risk for unfavorable outcomes.

Several severity scores have been developed, such as the Acute Physiology and Chronic Health Evaluation II (APACHE II) score,^(6)^ the mortality probability model,^(7)^ the Simplified Acute Physiology Score II (SAPS II),^(8)^ and more recently, the SAPS 3.^(9)^ Almost all severity scores use a group of demographic, clinical and physiological variables from the first day of the ICU stay to obtain an individual patient score and a prediction of in-hospital mortality. Typically, the abovementioned severity scores are used to monitor the performance of a single ICU, to adjust mortality of different ICUs to its case-mix and for helping to guide resource allocation.^(10)^ The currently available models are not useful and were neither designed nor validated for individual patient management.^(11,12)^ These scores were also not designed to evaluate post ICU discharge prognosis.^(2,7,13-16)^

Some investigators advocate that the pro- or anti-inflammatory status of the patient could be used as a potential risk factor upon ICU discharge.^(17,18)^ Biomarkers, such as C-reactive protein (CRP), procalcitonin (PCT) and lactate, have been studied with respect to hospital and ICU outcomes with conflicting results.^(19-21)^

Systemic levels of soluble urokinase-type plasminogen activator receptor (suPAR), a protein derived from cleavage and release from neutrophils, lymphocytes, endothelial and malignant cells, has recently been recognized as a potential prognostic biomarker of infectious disease.^(22)^ Various studies have been conducted on suPAR, the majority of which have focused on the ability of suPAR to predict sepsis and mortality in patients with bacteremia, systemic inflammatory response syndrome, sepsis, and septic shock.^(23-26)^ Systemic levels of suPAR have been found to be significantly higher in critically ill patients who exhibit poor outcomes.^(27)^ The role of suPAR as a prognostic marker of hospital mortality after ICU discharge has yet to be evaluated. Systemic levels of suPAR remain elevated long after clinical recovery, declining only after several weeks.^(28)^ Therefore, suPAR appears to be a promising prognostic marker in critically ill patients.

The aim of our study was to determine the predictive value of suPAR in the assessment of outcome (hospital mortality) of patients discharged alive from the ICU.

## METHODS

We conducted a prospective, single center, observational study over 24 months (June 2011 - June 2013) at the ICU of *Hospital de São Francisco Xavier*, an 8-bed multidisciplinary ICU.

The local Ethics Committee approved the study design, and informed consent was obtained from all patients or legal representative before study inclusion.

All patients discharged alive from the ICU were included, except for those with age < 18 years, those transferred to another ICU, and those with a do not resuscitate order.

Patients were followed until hospital death or hospital discharge.

Patient survival at 28 and 90 days after ICU discharge was also analyzed.

Data collected included admission diagnosis and past medical history. Vital signs were evaluated hourly, and daily extremes were recorded. APACHE II was calculated 24 hours after ICU admission.

C-reactive protein levels and white cell count (WCC) were measured at admission and daily until discharge. suPAR levels and SOFA scores were collected upon ICU discharge.

Measurement of CRP was performed using an immunoturbidimetric method (Tina-quant CRP; Roche Diagnostics, Mannheim, Germany).

suPAR was measured using a venous blood sample collected into an EDTA tube, centrifuged and frozen at -80ºC. Measurements were performed in duplicate using an enzyme-linked immunosorbent assay (suPARnostic^®^, ViroGates, Lyngby, Denmark) following the manufacturer's instructions. The lower limit of detection was 1.1ng/mL.

Subgroup analysis was performed in patients with sepsis diagnoses. Sepsis was defined according to 2001 international consensus definitions.^(29)^

### Statistical analysis

Data are presented as the mean ± standard deviation (SD). Categorical variables are presented as rates or percentages. Comparisons of parametric variables between groups were performed with an unpaired Student's *t*-test, and nonparametric variables were compared between groups using a Mann-Whitney test.

To compare the predictive value of the biomarkers and severity scores, receiver-operating characteristic (ROC) curves were built and the area under the curve (AUC) was determined. DeLong was applied to determinate the statistical significance of the differences between the AUC values.

The primary outcome variable was post ICU mortality.

To study the effect of biomarkers and SOFA on mortality, we used logistic regression. The unadjusted odds ratio (OR) and the corresponding 95% confidence interval (95%CI) were computed for each variable.

The level of statistical significance was set at 0.05 and all tests were two-tailed. We used the Statistical Package for Social Science (SPSS) statistical software package, version 19.0 (SPSS, Inc., Chicago, IL, USA) for all statistical analyses.

## RESULTS

A total of 202 patients (112 women and 90 men) were included, with a mean age of 65.3 ± 16.3 years and a mean APACHE II score of 22.0 ± 9.0. Post ICU hospital mortality rate was 14.6%, and hospital readmission rate was 38,4%.

Nonsurvivors were older and more seriously ill, with higher severity scores, and requiring more vasopressors than survivors. We did not find significant differences in admission diagnoses between groups. Clinical and demographic characteristics are presented in table 1.

**Table 1:** Baseline characteristics of patients

	All (N = 202)	Survivors (N = 173)	Nonsurvivors (N = 29)	p values
Age (years)	65.6 ± 16.3	64.3 ± 16.6	73.7 ± 12.2	0.004
Sex (M/F)	90/112	77/96	13/16	NS
APACHE II	22.0 ± 9.0	21.2 ± 8.7	26.9 ± 9.0	0.002
Admission diagnosis				NS
Respiratory	81	67	14	
Cardiovascular	33	25	8	
Renal	16	13	6	
Neurological	15	14	1	
Gastroenterological	10	9	1	
Surgical	9	8	1	
Trauma	6	6	0	
Metabolic	5	0	0	
Others	27	26	1	
ICU length of stay (days)	8.8 ± 22.4	8.7 ± 24.4	9.7 ± 8.8	NS
Hospital length of stay (days)	32.1 ± 35.3	29.8 ± 35.7	38.8 ± 32.5	NS
Sepsis	97 (48.0)	82 (47.4)	15 (51.7)	NS
Mechanical ventilation (days)	3.2 ± 6.0	2.9 ± 5.8	4.9 ± 7.8	NS
Renal replacement therapy (days)	1.4 ± 3.3	1.2 ± 3.0	2.8 ± 4.8	NS
Vasopressor (days)	1.1 ± 1.9	0.9 ± 1.5	2.3 ± 3.4	< 0.001

M/F - male/female; APACHE - Acute Physiology and Chronic Health Evaluation; ICU - intensive care unit; NS - nonsignificant. P value for comparison between survivors and nonsurvivors. The results are expressed as mean ± standard deviation, n, or number (%).

At ICU discharge, nonsurvivors were sicker, had higher SOFA scores (p < 0.001) and presented with higher suPAR levels (p = 0.003) than survivors. The other biomarkers (C-reactive protein and WCC levels) were similar between the two groups (Table 2).

**Table 2:** Biomarker levels and Sequential Organ Failure Assessment at intensive care unit discharge

	All (N = 202)	Survivors (N = 173)	Nonsurvivors (N = 29)	p values
suPAR (ng/mL)	7.7 ± 4.3	7.4 ± 4.1	9.9 ± 4.8	0.003
CRP (mg/dL)	7.1 ± 6.0	7.1 ± 6.1	7.3 ± 5.1	NS
WCC (x1000/mL)	10.5 ± 4,9	10.6 ± 5,0	9.9 ± 4.0	NS
SOFA	2.7 ± 1.7	2.4 ± 1.6	4.1 ± 1.3	< 0.001

suPAR - soluble urokinase-type plasminogen activator receptor; CRP - C-reactive protein; WCC - white cell count; SOFA - Sequential Organ Failure Assessment; NS - nonsignificant. The results are expressed as the mean ± standard deviation.

Among the studied prognostic variables, the best predictors of post ICU mortality were APACHE II (AUC 0.70) and SOFA (AUC 0.78). The ROC curve for suPAR yielded an AUC of 0.68 (p = 0.002), which was higher than the AUCs for CRP (AUC 0.54) and WCC (AUC 0.48).

The combination of suPAR with APACHE and SOFA increased predictive ability (Table 3). Despite the improvement in mortality prediction, predictive ability did not reach a combined sensitivity or specificity above 80%.

**Table 3:** Receiver operating characteristic curve analysis showing the prognostic power of biomarkers and severity scores in predicting mortality

Index variable	AUC	95%CI	p value
suPAR	0.685	0.586 - 0.785	0.002
CRP	0.536	0.423 - 0.649	0.538
WCC	0.476	0.365 - 0.586	0.679
APACHE II	0.699	0.606 - 0.793	0.001
SOFA	0.780	0.702 - 0.850	0.000
suPAR + APACHE II	0.721	0.630 - 0.812	0.045
suPAR + SOFA	0.803	0.734 - 0.872	0.000

AUC - area under the curve; 95%CI - 95% confidence intervals; suPAR - soluble urokinase-type plasminogen activator receptor; CRP - C-reactive protein; WCC - white cell count; APACHE - Acute Physiology and Chronic Health Evaluation; SOFA - Sequential Organ Failure Assessment. Discrimination is presented as area under the curve with 95% confidence intervals. DeLong analysis was applied to determinate the statistical significance of the difference between the areas under the curve. AUCs of different variables were compared to the AUC of SOFA.

Multivariate logistic regression analysis was performed with post ICU in-hospital mortality as the dependent variable. We included the following five variables in this model: APACHE II, SOFA, CRP, suPAR, and WCC (Table 4). SOFA was independently associated with a higher risk of in-hospital mortality (OR 1.673; 95%CI 1.252 - 2.234), 28-day mortality (OR 1.861; 95%CI 1.856 - 2.555) and 90-day mortality (OR 1.584; 95%CI 1.241 - 2.022).

**Table 4:** Odds ratios and confidence interval limits of biomarkers and clinical scores at intensive care unit discharge as well as 28 days and 90 days after intensive care unit discharge

Index variable	OR	95%CI	p value
suPAR	1.060	0.965 - 1.165	0.233
CRP	0.980	0.906 - 1.065	0.639
WCC	1.000	1.000 - 1.000	0.336
APACHE II	1.036	0.991 - 1.085	0.128
SOFA	1.673	1.252 - 2.334	< 0.001
suPAR*	0.987	0.887 - 1.098	0.786
CRP*	0.906	0.815 - 1.006	0.735
WCC*	1.000	1.000 - 1.000	0.023
APACHE II*	1.008	0.959 - 1.059	0.519
SOFA*	1.861	1.356 - 2.555	< 0.001
suPAR†	0.988	0.905 - 1.079	0.786
CRP†	0.988	0.921 - 1.060	0.735
WCC†	1.000	1.000 - 1.000	0.023
APACHE II†	1.014	0.972 - 1.058	0.519
SOFA†	1.584	1.241 - 2.022	< 0.001

OR - odds ratio; 95%CI - 95% confidence interval; suPAR - soluble urokinase-type plasminogen activator receptor; CRP - C-reactive protein; WCC - white cell count; APACHE - Acute Physiology and Chronic Health Evaluation; SOFA - Sequential Organ Failure Assessment;

* 28 days post intensive care unit mortality;

† 90 days post intensive care unit mortality.

Documented sepsis was present in 101 patients (50%). The presence of sepsis did not influence post ICU outcome, with similar mortality rates between septic and nonseptic patients. Similarly, to the general patient population, only SOFA score was associated with poor outcome and with a higher risk of hospital mortality (OR 1.876; 95%CI 1.238 - 2.842) (Table 5).

**Table 5:** Odds ratios and confidence interval limits of biomarkers and clinical scores at intensive care unit discharge in septic patients

Index variable	OR	95%CI	p value
suPAR	1.112	0.977 - 1.265	0.109
CRP	0.956	0.862 - 1.073	0.397
WCC	1.000	1.000 - 1.000	0.333
APACHE II	1.018	0.965 - 1.085	0.513
SOFA	1.876	1.238 - 2.842	0.003

OR - odds ratio; 95%CI - 95% confidence interval; suPAR - soluble urokinase-type plasminogen activator receptor; CRP - C-reactive protein; WCC - White cell count; APACHE - Acute Physiology and Chronic Health Evaluation; SOFA - Sequential Organ Failure Assessment.

## DISCUSSION

In this prospective observational study, we evaluated the performance of suPAR levels taken upon ICU discharge to predict post ICU mortality. Our data show that suPAR levels at ICU discharge are higher in hospital nonsurvivors.

In addition to the accuracy of suPAR in assessing the risk of post ICU mortality being lower than current severity scores, and its combination with these scores only slightly improved predictive ability for post ICU mortality.

Some investigators advocate that post ICU death is related to a persistent inflammatory response, with endothelial dysfunction and microcirculatory abnormalities present in nonsurvivors who have higher biomarkers levels.^(30)^

Various biomarkers have been proposed to be of potential use in prognostication. CRP concentrations have been extensively used and correlate with ongoing organ dysfunction, ICU mortality and likely also with bacterial burden.^(31-33)^ This marker is routinely measured in the ICU and has advantages of simplicity, reproducibility and speed.^(31,34)^

C-reactive protein has been studied as a prognostic biomarker for in-hospital mortality and readmission after ICU discharge.^(17,18,20)^ Because these results are seemingly conflicting, there is no evident consensus for using serum CRP and other biomarkers for post ICU prognosis.^(19,20,30)^

Recently, higher suPAR and pro-adrenomedullin (proADM) levels upon ICU admission seemed to be correlated to hospital mortality in septic patients.^(35)^ Similar to our data, in this study, prognostic accuracy was significantly better for severity scores than for any of the analyzed biomarkers. The best AUC for the prediction of in-hospital mortality was generated using APACHE II (0.82) and SOFA (0.75) scores. The ROC curve for suPAR yielded an AUC of 0.67, which was higher than those of proADM (0.62), CRP (0.50) or PCT (0.44). The combination of severity scores and biomarkers did not improve AUCs.

More recently, Jalkanen et al. studied a cohort of critically ill nonsurgical patients and found that low suPAR concentrations were predictive of survival.^(36)^ However, in that study, neither classical biomarkers nor severity scores were compared for the assessment of risk mortality.

Our study analyzed suPAR levels at ICU discharge. The biological characteristics of suPAR, which are only slightly influenced by circadian changes and remain stable in systemic circulation within the first days of a sepsis course, might explain its superiority over other biomarkers, namely, CRP and PCT.^(27)^

However, in our study, suPAR levels, despite being increased in hospital nonsurvivors, were not associated with higher risk of death either alone or in combination with severity scores. In addition, suPAR levels did not show any correlation with post ICU mortality in septic patients.

We found that a single determination of suPAR upon ICU discharge was a better tool for predicting in-hospital mortality than CRP. However, the prognostic accuracy was significantly better for APACHE II or SOFA scores than for any of the analyzed biomarkers. The combination of biomarkers with these severity scores only slightly improved their prognostic accuracies. Like other biomarkers, suPAR as a single biomarker is not a strong enough predictor for clinical decision-making.

## CONCLUSION

In the present study, we compared severity scoring systems and biomarkers for predicting mortality in patients discharged alive from intensive care units. Despite suPAR levels being slightly better than those of common biomarkers, including C-reactive protein, they did not exhibit superior performance than severity scores. At intensive care unit discharge, suPAR is a poor predictor of post intensive care unit prognosis.
